# Steam Pressure in Vulcanizers

**Published:** 1864-12

**Authors:** A. Lawrence


### STEAM PRESSURE IN VULCANIZERS.

**Read before the Massachusetts Dental Association, Jan. 2, 1865.**

BY A. LAWRENCE.

In the hope that a few "practical hints" upon the subject of steam, as applied in the vulcanizing process, may be acceptable to the profession I will endeavor to give them.

I may premise by suggesting that although the Dental profession are not expected to turn engineers, *en masse*, yet not only they, but all others who attempt to generate steam at high pressures, should do so with a full knowledge of the agent which they are producing. Presuming Dentists to be uninformed upon this subject, does not, necessarily, imply a want of professional education, for until within a few years there has been no necessity for research, or even a thought, in this direction.

The introduction of vulcanite, however, has not only revolutionized mechanical Dentistry, but compelled us to use steam, whether familliar with it or not; and vulcanizing is now done daily at a required pressure doubly sufficient to propel the largest steamship afloat.

It may be inferred from what has thus far been advanced, that we have to deal with a monster whose persistent efforts for liberty can be held in check only by the strongest prison walls, and the utmost vigilance.

To the further consideration of the subject it becomes important to enquire whether the vulcanizers, now most in use, are sufficiently strong to resist the force applied. From experiments made at the Franklin Institute it was found that the tensile strength of wrought copper one inch square at a emperature of 302° is 30,872 lbs., and at 392° only 27,154 lbs. Now it will be found, by mathematical calculation, that at 320° the usual vulcanizing point, it is about 30,000 lbs.

The published tables, however, give 34,000 lbs. as the tensile strength of wrought copper, but it must be borne in mind that the latter figures are based upon experiments at a much lower temperature than that under consideration. Most vulcanizers are now made of sheet copper one-sixteenth of an inch in thickness, and agreeable to the foregoing facts, have a tensile strength of 1,875 lbs.; and one 4 inches in diameter will not sustain a pressure of more than 150 lbs. per square inch, or a temperature of 363°.

Let us next ascertain what force of steam is exerted upon the boiler, within a short range of temperatures. We find by the tables of Haswell, King, and others, that at 320° the pressure is 85 lbs., at 324° 90 lbs., at 328° 95 lbs., and at 332° it is 100 lbs. per square inch. These figures I have verified by a steam guage connected with my own vulcanizer, and which I now use in preference to the thermometer, as I consider it more convenient, safer, and less liable to accidents.

Practical engineers concur in the opinion that a force of not over one-half the sustaining capacity of the boiler can be safely applied.

Now then, if vulcanizers of the diameter previously given, were made of sheet copper one-eighth of an inch in thickness, they would be capable of sustaining a force of 298 lbs. per square inch, and, at the temperatures attained in practice, might be regarded as comparatively safe. Of course it must be understood that the thickness of metal should increase with the diameter of the boiler, when designed for a given pressure.

Having given the statistics, little remains to be said, except, perhaps, to call in question the expediency of jeopardizing our lives every day in the week for the paltry sum necessary to furnish a trifle more metal to our vulcanizers.

The cost of manufacturing, aside from the stock, would not be enhanced.

It may be urged that vulcanizer explosions are of very rare occurrence.

Admitted; but therein consists the wonder, for it is not surprising that a few are "blown up," but that more or all are not.

The vulcanizer is nothing more or less than a small boiler for generating steam; and in its use we should so regard it, for the fact it is small does not deprive it of liability to the same accidents, or some of them at least, that larger ones are.

We recoil with a certain degree of apprehension from the presence of the large marine boiler carrying from 20 to 40 pounds of steam, while we sit down before the small vulcanizer with as much non chalance as to a cup of old Hyson. The difference is only as between the 100 pound Parrott gun and a pocket pistol.

Steam is easily managed so long as it *is* managed, its control being dependent upon certain well established principles, a departure from which can not safely be indulged in unless, indeed, one wishes to enjoy the extreme felicity of an aerial journey at the expense of all earthly ties.

However agreeable it might be to others, to see their own heads kiting through the air, I must confess that I have no unconquerable desire to participate in any such amusement.

I do not by any means wish to be regarded as an alarmist, but there is, most certainly, a limit to the resisting capacity of the boilers we use, as is the case with those intended for other purposes.

Suppose the bulb of the thermometer gets slightly fractured and the accident not being discovered, the vulcanizer is put to use, what then?

If the damage is slight, the mercury may still be made to rise in the tube at high temperatures, but will not truly indicate the full heat or force within. Some time ago I had some difficulty in producing a desirable shade in my vulcanite work; it was too dark, as is the case when overheated, and I came to the conclusion that the gum had deteriorated in quality.

Other samples of gum were tried, and at varying lengths of time, yet with the same result.

No defect could be discovered in the thermometer by the naked eye, but a microscope revealed a slight crack in the bulb, and the mystery was solved. But what force of steam was produced during these almost despondent trials?

Although my vulcanizer would safely bear a pressure of 100 pounds per square inch, I concluded to use a steam guage for the future, and now feel a security in its use positively refreshing. The guage I use is that manufactured by the American Steam Guage Co., No. 44 Exchange St. Boston.

Considering the further continuation of these remarks undesirable at this time, I leave the subject to the reflection of any who may think it worth their attention.

